# Perceived harms and protective behavioural strategies among khat chewers: a qualitative study in Jimma, Ethiopia

**DOI:** 10.1186/s12954-023-00890-y

**Published:** 2023-10-24

**Authors:** Amanti Baru Olani, Tom Decorte

**Affiliations:** 1 Institute for Social Drug Research, Department of Criminology, Criminal Law and Social Law, Ghent University, Universiteitstraat 4, 9000 Ghent, Belgium; 2 Department of Sociology, Jimma University, Jimma, Ethiopia

**Keywords:** Khat, *Catha edulis*, Protective behavioural strategies, Harm reduction, Harms, Health effects, Socio-economic effects, Ethiopia

## Abstract

**Background:**

While there have been many previous studies focusing on the adverse effects of khat chewing, attempts to investigate the protective behavioural strategies (PBS) employed by the khat using population are rare. PBS are strategies that substance users employ to minimize or alleviate the possible negative consequences related to the behaviour. This study focuses on the harms that chewers associate with khat use, and the behavioural strategies they practise to prevent or minimize these harms.

**Methods:**

A community-based qualitative study was conducted using a snowball sampling technique to recruit a diverse sample of khat chewing participants (*N* = 102) in Jimma city, Ethiopia. Face-to-face in-depth interviews were carried out with the participants.

**Results:**

Participants identified a variety of harms likely to result from chewing khat. These include impacts on their finances, work, social life and health. The PBS that participants employed to avoid or minimize the risks were classified into four themes based on their temporal sequence with khat chewing sessions: prior to chewing, during chewing, after chewing and general PBS covering the whole of their khat chewing career. The PBS enable khat chewers to prevent or minimize the adverse health consequences of chewing, socialize and work without or with fewer difficulties and manage their economy successfully.

**Conclusion:**

The study participants believe that khat-related harms are avoidable if khat users implement appropriate strategies prior to, during and after chewing, and if they apply PBS to *khat-related factors* (e.g. type, amount and frequency), *set factors* (e.g. reason for using and health behaviour) and *setting factors* (e.g. place of use, when used, with whom used and social norms) covering the whole of their khat chewing career.

## Introduction

Khat is a plant whose young and tender leaves are chewed for their stimulant effects in the East African countries, the southern part of the Arabian Peninsula [1, 2] and in the West among the diasporas from these regions [3, 4]. The plant contains several chemicals, although cathinone (schedule I stimulant) and cathine (schedule IV stimulant under the Controlled Substances Act) are the main causes of the euphoric effect that khat chewing provides, which is comparable to amphetamine use [5]. In traditional khat chewing areas like Ethiopia, khat is an integral part of long-established socio-cultural traditions and economic activities [5, 6]. Moreover, there is evidence of the medicinal use of khat for health problems such as headaches, cold, arthritis, fevers, depression, erectile dysfunction, malaria, high blood pressure and mouth swellings [5, 7].

Khat is a controversial substance due to its cultural and socio-economic significance on the one hand and concerns about its possible negative health impacts on the other. Although limited use of khat has not been found to have any accompanying serious mental health consequences, long-term use may lead to psychiatric disorders ranging from minor reactions to the development of psychosis [2, 8, 9], and it may cause impairment to the different dimensions of cognitive domains such as working memory, decision-making and visual memory [10]. Numerous physical health problems have also been associated with khat use, and, as with mental health, adverse effects are commonly linked with prolonged or excessive use, although the mechanisms of action are not well elucidated in most cases. Khat chewing is significantly associated with multiple sexual partners [11], spermatorrhoea (involuntary sperm leakage), decreased libido, impotence and lower sexual performance [12], delivery complications during childbirth and lower birth weight [12–14], liver injury [3] and premalignant oral lesions [15–17]. It is also feared that cancers of the digestive system, kidney failure, poisoning and death may result from consuming pesticides used on khat, since khat leaves are normally chewed fresh without being washed or thermally treated [18]. Socially, khat use is reported to cause adverse consequences such as loss of relationships with children, weakening of family relations, divorce and damage to the family economy, especially among people who are poor [2, 19].

The previous studies of khat use have mainly focused on the negative aspects of chewing, and attempts to investigate the protective behavioural strategies (PBS) employed by the khat chewing population are scarce. This lack of attention to PBS may be influenced by the common stereotype that drug users are not concerned about the possible adverse effects accompanying their behaviour [20] and the idea that khat use is usually impulsive and unplanned. However, the use of any substance may involve behavioural strategies capable of protecting people using it from the potential negative consequences of its use. Protective behavioural strategies, alternatively called behavioural self-control strategies [21] and harm reduction practices [22, 23], are behaviours that substance users employ to minimize or alleviate the possible negative consequences related to their behaviour [24]. Studies on PBS are now receiving increasing international attention, since it is believed that if users are aware of how best to use substances, and carefully control why, when, where and with whom they use them, the associated risks can be significantly reduced [25]. Some of the PBS related to alcohol, for instance, include setting personal drinking limits, avoiding situations that can lead to drinking too quickly, and making safe travel plans for when drunk [26–28]. In the previous studies on alcohol, protective behavioural strategies were associated with the consumption of fewer drinks [29] and with fewer alcohol-related adverse consequences [24, 27, 30].

In contrast with studies that assess individual characteristics which are difficult or impossible to modify, such as gender and ethnicity as risk factors, PBS-based research focuses on cognitive and behavioural aspects that can be modified through intervention. Furthermore, PBS-based studies identify and promote responsible use rather than abstinence or the elimination of substance use [26]. Research into PBS, therefore, has the potential to minimize harms among those for whom abstinence is not their primary goal, which could be the case for users of social drugs such as khat. In the context of khat use, understanding the PBS that people use is important in order to contextualize future interventions that aim to reduce the risks. This study addressed that rationale through a qualitative, community-based study with khat chewers in Jimma. It explores the perceived harms that chewers associate with khat use, and the behavioural strategies they practise to minimize them.

## Methods and sample

### Description of the study area

The study was carried out in Jimma, a city in Oromia regional state, Ethiopia, 352-km south–west of the capital city Addis Ababa. Jimma is one of the main centres of production and consumption of khat in Ethiopia. Khat consumed in the city is cultivated in the surrounding rural villages and sold in numerous khat retail sites in the city.

### Study design

This study is a part of a broader qualitative project exploring the self-regulation of khat use. This section of the fieldwork investigates the protective behavioural strategies (PBS) used by khat chewers. A qualitative approach was employed to generate a detailed understanding of chewers’ perception of khat use risks and the harm reduction strategies they employ. The fieldwork was conducted in Jimma over a 10-month period from May 2021 to February 2022.

### Sampling procedure

The sample was drawn from the general community, since the intention was to investigate the PBS that khat chewers *naturally* implement. The study employed the principle of maximum variation sampling since it offers two advantages. First, this sampling strategy helps to document uniqueness related to PBS; and second, it can identify important shared patterns in PBS that cut across cases [31]. The literature review and preliminary field observations demonstrated that a diverse range of people is involved in khat chewing, varying in terms of their wealth, education, age, gender, ethnicity, place of residence and so on. To enhance the diversity of the sample, each participant was asked to list as many nominees from their own network as possible, and subsequent participants were selected purposively, so that the key dimensions of all the variations listed above were represented. In total, 102 khat chewers took part in the study.

### Methods of data generation

The study relied on a semi-structured interview guide to generate a deeper understanding of chewers’ perspectives and experiences related to PBS in khat use. Khat chewers were specifically asked about their perception of the negative effects of khat in general, the negative impacts they faced and the risk reduction techniques they employed. The questions were initially broad, since there is no previous study to inspire more specific questions. As sampling and interviewing progressed, the questions became more specific and focused on elaborating PBS identified during the earlier interviews. The interviews lasted between 1 and 2 h 30 min.

The inclusion criteria were: being at least 18 years old and having used khat for at least 2 years. Additionally, the study focused on *current* khat chewers, which is usually defined as having used khat at least once in the month preceding the interview [32, 33]. Every individual who met the inclusion criteria was approached and informed about the study and the duration of the interview. Interviews took place ‘in an atmosphere where the subject felt relaxed, at a location where the interview is not easily disturbed, and under conditions that are preferred by the respondent’, and where friends, partners or acquaintances could not overhear the conversation [34].

### Data analysis

Interviews were audio recorded, transcribed verbatim and translated from Afaan Oromoo and Amharic into English. The data generated through the in-depth interviews were analysed thematically. Data coding and analysis were supported with NVivo software. The process of data analysis involved generating codes after reading all of the interview transcripts, sorting each idea in the interviews into the codes, revising the codes if necessary and, finally, developing the codes into themes based on their similarities.

### Ethical considerations

The study was approved by the Ethics Committee of the Faculty of Law and Criminology of Ghent University (Belgium). Before conducting the interviews, informed consent was received from all participants for both the interviews and audio recording. Information collected from the participants was stored securely and anonymized.

## Results of the study

A total of 102 khat chewers participated in the study. About 72.5% of the participants were male. The mean age was 31 years. In addition to students and unemployed individuals, participants were involved in a variety of occupational sectors including government offices, private businesses, daily paid employment and sex work. This section presents the findings related to the perceived harms of khat use identified by the study participants, and the PBS they employed to address them.

### Price instability

The study found that the price of khat varies depending on the seasons, weather and time of day. For instance, its price increases during the dry seasons when less is available and from 1 to 3 pm since demand is higher. Many of the participants stated that they do not use a fixed quantity of khat as they reduce the amount when the price goes up or when they feel their khat use is affecting their budget.

Coping strategies to deal with price surges include: joint buying (*giche*), since there is a minimum price below which retailers do not sell khat; buying later in the day, when the price falls; chewing slowly (*mabkakat*); chewing without sugar, since sugar increases the solubility of khat and makes chewers finish earlier; smoking cigarettes and/or drinking coffee while chewing and re-conceptualizing the reason why they chew khat, for instance to avoid withdrawal or to pass the time rather than for *mirkana.*^1^

I usually spend 20 birr per day on khat. With this money, I cannot buy khat up to 3 pm. You need to have more money to buy khat at 1 or 2 pm. But after 3 or 4 pm you can buy khat for 20 or 30 birr (female, 34 years old).

When the price of a particular type of khat increases, many chewers change their selection and purchase a cheaper variety. When chewers are short of money, they may rely on khat chewing friends and/or retailers to supply them.

### Dependence and tolerance

Most khat chewers recognize the potential to become dependent on khat chewing. As a result, they practise reflective thinking in which they check their condition and make decisions that will enable them to avoid or reduce dependence. Two reactive strategies for reducing dependence have been identified: the *khat holiday technique* and a *slow reduction technique*. Those adopting the *slow reduction technique* want to avoid the withdrawal effects associated with taking an abrupt break.

If I continuously chew khat from Monday to Sunday, I may not be able to stop it automatically on the next Monday. That gives me nightmares. What I do to avoid this is chew a small amount on Monday, and then chew only two or three sticks of khat on Tuesday, and then dependence goes away. When I am free, I can chew continuously for a week. The dependence then comes back. Using the same technique, I’m able to overcome it yet again (male, 40 years old).

Those using the *khat holiday technique* usually do so when they have experienced a strong negative reaction to the way they have been using khat. For instance, they were involved in heavy chewing and/or drinking on the previous day and need to take break (recover from the fatigue and harms caused to the body), or they notice that they are developing tolerance.

When you use khat the next day after jumping for a day or days, you will get nice *mirkana*. You taste the khat. I also know many other people who stop using for some days when they realize that their khat intake is increasing. When getting back, they will be able to reduce their amount because they can get satisfaction from a small amount (male, 28 years old),

Khat chewers borrow terminology from other fields, for instance car repairs and soccer, to describe stopping chewing. Terms used include *service megbat* (renewing a vehicle), *doro tera* (the place in Jimma where public minibuses park when off-service) and *gudat lay negn* (exhaustion, especially those combining khat with alcohol)*.* During their ‘holiday’ from chewing khat, users avoid khat chewing friends and places where it is sold, to avert developing a craving. They watch movies, spend time with friends who do not chew, concentrate on work and ‘eat whatever is available’ to recover. Khat chewers’ appetites are generally reported to be at their peak when on a khat holiday.

The proactive strategies employed to limit the development of *tolerance* to khat include limiting the amount used, not chewing continuously and limiting chewing sessions to the afternoon.

### Withdrawal

Many khat chewers report that they experience symptoms of withdrawal when they fail to use khat at the time they usually start chewing, which is mostly in the afternoon at around 1.30–3 pm. The withdrawal symptoms include yawning, feeling tired, irritable, sleepy, exhausted or restless, teeth-grinding and nightmares. Withdrawal can happen when khat chewers have no access to khat due to *external factors* such as economic pressure, work engagements or participation in social life, or *internal factors* such as feeling unwell or the need to reduce dependence.

Three categories of strategies are employed to cope with withdrawal symptoms: *distracting activities*, *denial* and *limited access*. Distracting activities include practicing alternative behaviours such as taking a walk, keeping busy, visiting relatives/friends, consuming caffeinated drinks and taking naps. Denial involves recognizing the feelings as withdrawal symptoms and not doing anything about them. Limited access is the practice of chewing only a small amount to avoid or minimize the withdrawal symptoms. Once their regular khat chewing starting time has passed, which is usually after 3 pm, most chewers no longer feel any withdrawal symptoms. Most believe that withdrawal symptoms are less serious when they are engaged in work, indicating the role of a person’s *setting* or environment in determining the degree of withdrawal and craving.

The existence of a strong tradition of khat sharing has helped khat chewers to get some khat for free from their friends, retailers or even strangers when they do not have money to buy it themselves. This tradition can be easily observed in public places and on public transport, where people with khat might even invite strangers to chew. Most khat chewers state that ‘access to khat is very easy’, and in this regard, a 43-year-old male khat chewer said, ‘If you are in Jimma, you don’t have much worry about accessing khat even if you have no money’.

Although the possibility of committing crime to access khat is less reported, a few khat chewers have described instances in which they have ‘offended others’ by committing thefts, and they usually explain this in the context of polysubstance use or the need to get an adequate amount of khat. A few participants also reported hustling for money or borrowing, usually giving reasons unrelated to khat, in order to access khat.

### Sleeplessness

The disruption of sleeping patterns due to khat chewing is a problem that participants frequently mentioned. To avoid sleeplessness, several strategies are employed, both *during* and *after* chewing.

*During* chewing, strategies include limiting the amount of khat, stopping chewing earlier and avoiding the consumption of caffeinated drinks although there are also many khat users who usually drink coffee while chewing, and often the coffee-making ceremony is an integral part of the khat chewing sessions.

The strategies employed *after* chewing include carrying out household chores (especially among women) to induce tiredness, taking a bath, reading, watching movies, drinking milk or non-caffeinated soft drinks or soup, eating a substantial meal and drinking alcohol. Although not prevalent, some chewers smoke cannabis after using khat to break *mirkana*.

The implication is that khat chewers have their own personal repertoire of techniques that they employ to prevent khat from affecting their sleep.

### Work

The popular portrayal of khat chewing is that small groups of friends gather in the afternoon to spend time ranging 4–6-h chewing khat at home, while taking off their shoes, wearing comfortable clothes and relaxing on a mattress. The implication of this stereotype is that the chewing of khat has a negative impact on people’s work.

Chewers use a range of strategies to successfully balance their khat chewing and their work. Some, especially those who are self-employed, prefer to chew khat while they are working (they chew at their workplace). Those who are government employees make *lulu*.^2^ A male khat chewer who works as a broker without a physical office explained his behaviour of combining work and chewing as follows:

When I chew khat, I do not like chewing in places like this one [the interview took place at his home]. I want to use it at my workplace [streets around the bus station]. You have seen me last time [I saw him moving around, holding khat]. While using khat I move here and there. There is work, there is brokering. You make money while chewing. When I use khat, it gives me energy; it stimulates me for work. In my life, I do not like chewing if I do not get money out of it (male, 34 years old).

Khat chewers involved in laborious occupations indicate that they feel energetic and motivated while simultaneously chewing and working. Among these participants, the chewing of khat itself is not reported to have a negative correlation with work; it is rather the withdrawal effects when khat is not accessible that could decrease their work performance and motivation. Understanding this performance-enhancing role of khat, employers in activities such as construction and furniture making usually provide khat for their employees in the afternoon. Participants who believe that the nature of their work does not allow them to chew while on duty, for instance teachers and public servants, only increase their khat intake during their free time at weekends. Increasing khat chewing at the weekend is accompanied by making adequate preparations such as eating enough food and having money for alcohol to break *mirkana*. Students state that chewing a large amount of khat makes them over-stimulated and unable to concentrate, and that is why they limit the amount they use.

Although most chewers report stopping chewing at around 6 or 7 pm, those who report chewing after that time indicate that they stop chewing earlier if they have to get up early the next day for work. Many users also state that they do not chew at night since it disturbs their sleeping patterns, and they generally chew in the afternoon rather than the morning, since they believe that it is better to dedicate the morning session to work. Chewing khat in the morning is considered to distract people from work and expose them to a high level of dependence. Chewing khat with friends who have a positive attitude towards work is another strategy used to reduce the impacts of chewing on work.

### Social life

Although khat is widely used in Jimma, there are norms of appropriate use whose violation would have a negative impact on the social life of chewers. *Where* to use khat is a part of the protective behavioural strategies that participants employ to avoid the social consequences of chewing. For instance, chewers who have a high social status, such as public servants and teachers, stated that they could be labelled as a bad person (*duriye)* and their social position ruined if they were to chew khat in public. Due to the need to conform to gender norms and to avoid being labelled deviant, many female participants also indicated that they do not chew khat in public.

I do not chew khat on the street. It is disgusting for a woman to chew khat on the street. People look down on you (female, 23 years old).

Khat chewing is usually considered a factor that leads to divorce and family disintegration when men fail to spend adequate time with their marital partners because they are chewing khat at their friends’ homes, coffee shop or khat room. Some users chew khat at home for this reason, and the marital bond is assumed to be enhanced if the wife also chews khat or approves of the behaviour. In addition to chewing, the role of women includes preparing a coffee ceremony that gives ‘a special flavour’ to the khat chewing session. Not chewing khat in front of children is a harm reduction technique that some khat chewers employ, although this is different in the case of Muslim khat chewers, who usually give their children as young as 10 or 12 years old *a prayer khat*.^3^

Although there are those who prefer to chew alone for purposes like studying, the majority of participants discourage chewing alone, and they point out reasons like the role of khat in strengthening social relations and exchange of information when chewing in company.

### Gastrointestinal system, dental health and appetite

The physical health impacts most chewers associate with khat include effects on the gastrointestinal system, the teeth and appetite. Detailed discussions with many khat chewers reveal the belief that the chewing of khat is not in itself harmful to health if people eat enough, if other substances are not used and if the use of additives such as peanuts and sugar is avoided.

Constipation^4^ is one of the health impacts associated with chewing khat, and chewers minimize this risk by drinking plenty of water while chewing. Most khat chewers keep a bottle of water in front of them and sip it frequently while chewing. In addition to preventing mouth dryness while chewing, drinking water is also believed to have a protective effect against possible kidney complications among those who spend long hours sitting and chewing khat. Additionally, access to water while chewing is considered potentially lifesaving, as choking from khat is deadly. During the interview sessions, we observed several choking incidents when chewers’ eyes turned watery as they chewed. They coughed repeatedly and sipped water to displace the pieces of leaves trapped in the trachea. Some of the participants reported anecdotes they had heard about death resulting from choking on khat.

Many participants stated that they have changed their khat chewing behaviour to minimize risks as they have become more experienced in its use. A 37-year-old man, for instance, said, ‘I stopped using khat with sugar after 10 years of chewing. I was not conscious that the use of sugar could affect my teeth’. The use of a plenty amount of additives such as sugar and peanuts is common practice during the initiation phase of khat chewing, to alleviate the bitter taste of khat. However, many chewers report that they avoid additives, especially sugar, due to effects on a range of health issues including dental health, undesirable *mirkana*, khat-induced bezoar (blockage of gastrointestinal tract), gastric health, spermatorrhoea (involuntary semen leakage) and heavy sweating at night.

To avoid the possible physical health complications that could result from the use of khat, chewers adopt protective behaviour like eating a large meal before chewing. Those khat chewers who say that they have eaten adequate and ‘quality food’ usually report that their chewing is non-problematic and safe. Chewing khat without eating food is universally considered to be ‘unacceptable’ by users. They generally state that ‘khat is something that comes only after having food’, and reactions such as feeling dizzy, nausea and gastric problems are experienced when chewing without eating. A 45-year-old man described the risks of not eating food before chewing as ‘let alone without eating, khat is even dangerous when good food is not taken’, and for many participants, the amount and type of khat chewed depend on the quantity and quality of food eaten: ‘If I have eaten good foods like meat, I could chew a lot. Less quality foods like *shiro*^5^ doesn’t allow me to chew more’ (male, 28 years old). It is important to note that there is an exception to this, as some users chew khat to reduce their appetite for food, to deal with hunger due to poverty and even if they cannot afford the food they perceive as being good quality.

Although high importance is attached to eating enough before chewing, many khat users have less appetite for food after chewing. Specifically, most of those who are accustomed to drinking alcohol after chewing have little appetite for food, and most reported that they drink alcohol but do not eat after a khat session. Accordingly, many of the khat users who are not able to eat food after chewing khat try to eat an adequate lunch before they start a chewing session. The techniques used to overcome appetite loss include taking a break of about 1 or 2 h between the ‘spitting of khat’ and dinner time; and selecting juicy and simple foods instead of ‘hard’ and spicy ones.

Most khat chewers agree that when khat is used in moderation, the risks associated with its health impacts are reduced. For instance, since chewing khat while also smoking cannabis or drinking alcohol increases khat consumption, some of the khat users indicate the need to avoiding such combinations:

We call it writing and overstriking (*serez-delez)* when you smoke cannabis while chewing. When you smoke you feel depressed, and then, you need more khat to get stimulated again. You start to chew afresh. That way, you chew a lot and you need more alcohol to break the *mirkana* (female, 24 years old).

Limiting the amount chewed is related to the need to avoid khat’s impact on the gastrointestinal system, since consumption of large amounts is reported to have resulted in gastric health problem for some people. Many chewers believe that drinking milk, fenugreek juice or flax seeds juice are beneficial when experiencing digestive discomfort, although several khat chewers said that they were too poor to afford these products.

Although khat is widely associated with dental health effects, many chewers disregard the direct connection and instead associate it with the use of additives or cigarettes. While some of the PBS employed to avoid dental decay and discolouration are common, for instance rinsing the mouth with water after chewing and brushing their teeth, usually with a stick from the khat tree, there are also less common strategies, for instance cleaning the teeth with a paste made of garlic, ginger and salt, and rinsing the mouth with urine.^6^

### Sexual performance

Although there was no clear opinion on the relation between khat chewing and sexual performance, many chewers believe that the use of khat reduces their desire for sex and/or leads to premature ejaculation. Although more men than women acknowledged the effects on sexual performance, both sexes believe that khat has negative impacts. Female participants complained that men are usually not interested in sex after chewing khat, unless they have drunk alcohol. Male khat chewers employ techniques like avoiding a khat type that would reduce sexual performance, drinking alcohol after chewing, not chewing prior to a date, stopping chewing early—for instance at 4 or 5 pm—on the days when they are planning sexual intercourse at night, eating enough after chewing and delaying having sex until the following morning.

The other sexual health effect of chewing khat is spermatorrhoea (involuntary sperm leakage), although very few participants reported this problem. Opinion is divided on the cause, as some chewers associate it with peanuts, others associate it with chemically sprayed khat and yet others state that it is an effect of khat itself. The protective behavioural strategy used in this regard differs accordingly, and it ranges from avoiding the use of peanuts and chemically sprayed khat, to chewing in moderation. Many khat chewers claim that they can identify chemically sprayed khat by its shiny and sticky leaves.

### Mental well-being

Concerning the mental health impacts of khat, beliefs were very much divided among the participants but all of them agreed that most risks are avoided by using khat in the appropriate way. For instance, mixing different khat types during a given chewing session is believed to cause stress, so this is avoided. Many khat chewers also prefer to chew khat in the company of others as they believe that they are more likely to experience stressful thoughts and hallucinations when chewing alone, while chewing with others is regarded as relaxing.

The other strategy involves only using khat when in a good mood, or resolving stressful issues before starting to chew.

If I am not in a good mood, before starting chewing khat what I usually do is stabilize myself, for instance by listening to music. If you start chewing khat while you are in a worry, khat exposes you to more stress (male, 36 years old).

Many khat users also state the importance of chewing with friends who do not talk too much, as talking is considered disruptive to *mirkana* time. Some participants prefer to use at home or in a peaceful and quiet location, since if they are disturbed during *mirkana*, they are likely to develop a headache or confusion. Other strategies used to prevent possible effects on their mental health include selecting the right type of khat, not chewing a variety that is not fresh^7^ and avoiding chewing in the morning and afternoon of the same day.

## Discussion

This study is a first attempt to focus on the protective behavioural strategies (PBS) employed by khat chewers. We found that khat users employ a wide variety of behavioural strategies with the rationale of preventing or attenuating the possible negative consequences of chewing. The belief about the negative effects of khat use is widely shared among the chewers, although there is variation concerning which behavioural pattern of chewing constitutes risk, the type of risk identified and, as a result, the nature of PBS employed. The strategies employed can generally be classified into four categories based on their temporal sequence with khat chewing sessions: PBS prior to chewing, PBS during consumption, PBS after chewing and general strategies covering the whole of their khat chewing career (see Table 1). Although the protective behavioural strategies employed in khat use are to a great extent different from those employed for other substances, the temporal occurrence of such strategies is also reported in other substances, for instance as preloading and postloading in ecstasy [36], before, during and after a chemsex session [37] and before, during, after and instead of using marijuana [38]. In the case of ecstasy, for instance, pharmaceuticals and natural products are used both before (preloading) and after (postloading) ecstasy use to reduce risks such as brain damage [36].

**Table 1:** A summary of thematized PBS employed by khat chewers

	PBS employed	Targeted risk
General PBS	Limiting the amount of use Buying khat when it is cheap or buying a cheaper type Chewing while also working Avoiding the need to chew khat for *mirkana* Obtaining khat from friends or retailers for free	Economic impacts
	Slow reduction Taking a break from chewing for a while (khat holiday) Engagement in other activities instead of chewing Limiting the amount of use Using khat only in the afternoon	Dependence and tolerance
	*Distracting activities* such as going for a walk, busying oneself with activities, visiting relatives/friends, consuming caffeinated drinks and sleeping at their usual khat chewing time Acknowledging the withdrawal symptoms and not doing anything about them *(denial)* Chewing a small amount of khat *(limited access)*	Withdrawal symptoms
	Chewing while also working, if the nature of the work allows Chewing quickly (making *lulu*) before work or chewing after work or only during free time, if the nature of the work does not allow, and chewing while also working if the nature of the work allows Limiting the amount of khat chewed Avoiding chewing at night so that it does not disturb their sleeping pattern and they will be able to get up on time for work Avoiding the need to chew in the morning and dedicating the time wholly to work Chewing with friends who have a positive attitude towards work	Work
	Avoiding the use of other substances	Health
	Not chewing before a date	Sexual performance
PBS before the chewing session	Eating well, selecting the khat type convenient to their taste and health, avoiding buying chemically sprayed khat	Physical health
	Avoiding to buy a khat type thought to reduce sexual performance	Sexual performance
PBS during the chewing session	Selecting the right place for chewing Chewing in company to strengthen social bonds	Social impacts
	Avoiding additives like sugar and peanuts, using adequate water, limiting the amount of khat chewed, avoiding drinking alcohol or smoking cannabis as this increases the desire to chew and there may be related health effects	Physical health
	Stopping chewing ahead of time if they plan sexual intercourse	Sexual performance
	Limiting the amount of khat, stopping chewing earlier and avoiding the consumption of caffeinated drinks	Sleeplessness
	Avoiding mixing different khat types, chewing with others, avoiding stressful thoughts while chewing through listening to music or controlling their thoughts, avoiding exposure to noise or too much talk during *mirkana*	Mental health
PBS after the chewing session	Carrying out household chores to induce tiredness, taking a bath, reading, watching movies, drinking milk, non-caffeinated soft drinks or soup, eating a substantial meal and drinking alcohol	Sleeplessness
	Drinking alcohol, eating well	Sexual performance
	Taking an adequate break after ‘spitting khat’ and using juicy and simple foods to overcome appetite loss	Appetite loss
	Drinking milk, fenugreek juice or flax seeds juice to avoid impacts on the gastrointestinal system Mouth rinsing or brushing teeth to avoid dental health effects	Physical health

For most of the chewers, harm reduction strategies are a fundamental part of their khat chewing practices. The previous studies of khat chewers have failed to identify and capitalize on the harm reduction strategies that chewers already employed, and instead present them as naïve consumers who lack awareness of the harms of chewing and the need to quit [39–42]. Nevertheless, the perceived harms against which the protective behavioural strategies were used by particpants in this study are similar to those reported in the previous studies on khat [33, 39, 43, 44].

One of the PBS employed attempts to deal with khat’s price instability and prevent the negative economic consequences of chewing. Strategies to deal with adverse economic consequences include reducing the amount of chewing through delaying the time at which khat is bought and chewed, chewing slowly and smoking cigarettes or drinking coffee while chewing. Studies on other substances also indicate that users tend to reduce their consumption as a form of economic harm minimization. The difference is that, in the case of khat, price change is mainly nonlinear, and it varies across seasons and times during the day, unlike the effects of the top–down, intentional taxation and pricing policy system affecting substances such as alcohol and tobacco [45–48]. Accordingly, khat has an inbuilt system capable of preventing the tendency to continuously increase consumption, since chewers are forced to regularly adjust the amount they chew and the type of khat they prefer in order to reduce economic harms.

Similar to this study, the previous research has highlighted the dependence-producing potential of chewing [49–51]. The decision to reduce or avoid khat dependence usually results from reflective thinking about its impacts. The techniques used to overcome the risk of dependence are affected by the possible side effects of the chosen alternative, and the psychological or physical condition of the chewer. Generally, the *khat holiday technique* (for those who consider immediate change as preferable) and the *slow reduction technique* (for those who consider the withdrawal symptoms as painful) are *reactive* techniques used to reduce khat dependence once it has developed. Limiting khat chewing sessions mainly to the afternoon, rationing the amount of chewing—usually to one bag or less—and having a clear expectation for khat chewing (goal setting) are *proactive* strategies for avoiding dependence and tolerance.

Although craving is usually defined as the ‘irresistible urge’ for drug use [52], such a definition in the context of khat chewing is refutable as khat users have their own mechanisms for overcoming the urge to chew. The set of techniques at the disposal of khat chewing individuals and the khat sharing norm that enables chewers to obtain khat despite economic barriers significantly minimize the risk of involvement in crime as a means to access khat. This contrasts with evidence on illegal drug use, where the culture of sharing is minimal due to legal barriers or use is hidden and individualized through a fear of stigma, and where the likelihood of committing crime to access the drugs is higher [53, 54].

Most khat chewers recognize that chewing khat results in sleeplessness, which can lead to health problems (both mental and physical) and affect punctuality at work. Nevertheless, as khat is both a recreational and a performance-enhancing substance, whether sleeplessness constitutes a problem depends on the context of use. For instance, students and long-distance drivers intentionally use khat to stay awake at night. Several harm reduction techniques to avoid sleeplessness are employed both during and after chewing khat. Some of the techniques, for instance drinking alcohol, are the same as those employed to avoid the sleeplessness that follows the use of other stimulants [55]. Nevertheless, the (ab)use of sedatives, which is widely employed to treat sleep disorder after the use of other stimulant substances [55, 56], is not reported in connection with khat chewing. The choice of a harm reduction technique to protect oneself from sleeping disorder is to some extent influenced by the gender and religion of the chewers. Women usually prefer to carry out household chores to exhaust themselves, whereas men tend to go for a walk or drink alcohol. Since drinking alcohol is considered *haram* (things forbidden by Islamic law) among devotee Muslims, they prefer alternative techniques to the followers of other religions.

Although there is a popular stereotype that khat chewing sessions are a ‘waste of time’ [43, 57–59] since khat chewers are believed to take time off work to indulge in chewing, the strategies used by the chewers identified in this study challenge this. Khat use when not at work constitutes only one category of chewing pattern, and many users are able to reconcile working and chewing. Like most other drugs, khat use is regulated through informal social norms and rituals that influence who uses khat, where it is chewed, how and when. The violation of these norms leads to both the abuse of khat and negative societal reactions. These norms are mostly grounded in attempts to avoid khat’s negative impacts. For instance, chewing khat in the morning is socially disapproved of, and chewers also consider ‘morning khat’ (*ijabana*) to have a negative effect on their work, their appetite and the afternoon chewing session.

The health impact of khat has received more attention than any other issue in research studies and policy debates [5, 19, 60]. Khat chewers also recognize a wide variety of health problems that could result from chewing khat, and they employ PBS in an attempt to reduce the risks. Although these strategies are salient, they are usually based on users’ own negative personal experiences of khat rather than professional assistance or taking proactive measures. Concerning sexual performance, this study leaves unresolved the ongoing argument about whether chewing khat enhances, reduces or has no effect on sexual performance [12, 59, 61]. In a departure from the previous studies, however, it identified different PBS that chewers employ to minimize the risks.

In general, the results of this study lie in between the two extremes in the literature and policy debate concerning khat chewing where, on the one hand, it is considered ‘a benign habit’ that is equivalent to ‘drinking a couple of cups of coffee’ [62], and on the other hand, as represented in a large body of the literature, as a lethal drug with multiple health and socio-economic effects (5, 63). This study confirms that khat chewing involves risks, but it finds that chewers protect themselves through a variety of behavioural strategies and enjoy the positive aspects of the substance. The implication for practice is that the diverse PBS identified could be promoted by healthcare providers, the media, social workers and public policy-makers in order to reduce the possible negative consequences of khat chewing without necessarily coercing khat chewers to stop their use or criminalizing the use of the substance. Nevertheless, given that the findings are self-reported, such initiatives could only be implemented after conducting further studies to prove their effectiveness.

The community-based nature of this investigation and the diversity of the participants have revealed a wide variety of behavioural strategies that might help chewers to avoid the risks of khat use, and thus avoid the need for admission to treatment or serious effects on chewers’ socio-economic life. Since the study focused on khat consumption in the context of city life in Ethiopia, however, the results may not be generalizable to the population of khat chewers in rural areas and outside Ethiopian cities. The qualitative nature of the study also prevented the study from estimating the prevalence of the identified PBS and factors associated with implementing or failure to implement the strategies. Moreover, the effectiveness of the protective behavioural strategies to prevent the risks identified in the study needs further examination using prospective study designs.

## Conclusion

Participants of the study attributed several risks to their khat chewing behaviour. Risks identified include economic impacts, dependence and different forms of health problems ranging from physical ill health to mental disorders. However, khat chewers also believe that khat-related harms are avoidable if the chewer adapts their behaviour based on *khat-related factors* (e.g. type, amount, frequency and duration), *set factors* (e.g. reasons for using and health behaviour) and *setting factors* (e.g. place of use, when used, with whom used and social norms). Accordingly, khat chewers employ several protective behavioural strategies before, during and after khat chewing sessions, and during the whole time they are chewers, to reduce or prevent the possible negative effects of chewing. The identification of PBS in terms of their temporal sequence is indicative of the conscious decision-making process among chewers concerning khat use, and it challenges the conceptualization of drug use as a chaotic, compulsive and unconscious behaviour. The specific PBS employed among chewers is generally decided by their perception of harm, sociodemographic profiles such as economic status, gender and occupation, and the perceived side effects or effectiveness of the chosen harm reduction strategies.

## Data Availability

The anonymized interview transcripts used for the study are available upon reasonable request made to the corresponding author.

## Abbreviation

PBS: Protective behavioural strategies

## Footnotes

1 The term used to refer to the elated feeling or ‘high’ caused by khat consumption.

2 The practice of using khat in a hurry when there is not time to slowly chew and ingest its juice. In *lulu*, the entire teeth-digested khat leaves are swallowed, as opposed to swallowing mainly the juices (what is the usual procedure), using water to facilitate the swallowing. The word may have been derived from ‘*lulluqachuu’*, an Afaan Oromoo term for mouthwash. Some government employees make *lulu* for some 20 min between lunch time and the start of the afternoon work time, to limit the impact on work, as chewing large amounts takes too long, and others chew only a small amount after leaving work at 5 pm. However, the practice is usually associated with causing headache, stomach discomfort and high *mirkana*.

3 A prayer khat, locally called *caatii duwaayii*, is khat chewed by Muslims during prayer time, since it is believed that khat facilitates or strengthens the bond with Allah during prayer. A blessing is made on a prayer khat before starting chewing, and chewing such khat is believed to be taking the blessings. Everybody in the house is given the prayer khat, including children, although the amount given to children is very small.

4 On the other hand, there are khat chewers who report that they get the urge to defecate only when they have chewed, and otherwise, they experience constipation. Such people call the khat they use to overcome constipation *uddu-banaa,* which is an Afaan Oromoo term for *anal opener.*

5 A spicy stew made of powdered chickpeas. It is usually portrayed as a food of the poor.

6 The practice of mouth washing with fresh urine was reported by one participant, and he stated that some of his friends also employ similar protective strategies against dental decay and discolouration. This strategy was reported in the literature to have been practised in ancient Chinese medicine.

7 Khat is considered to be fresh only if it is chewed either on the day it is harvested, or if it has been harvested the previous day from the late afternoon onward. The *mirkana* from khat that is not fresh is believed to give rise to headaches and heartburn.
